# Male incontinence: Pathophysiology and management

**DOI:** 10.4103/0970-1591.32070

**Published:** 2007

**Authors:** Ajay K. Singla

**Affiliations:** Department of Urology, Wayne State University, USA

**Keywords:** Incontinence, prostatectomy, treatment

## Abstract

Post-prostatectomy incontinence in men is a devastating condition. It impacts the quality of life profoundly. Various types of male sling procedures have been introduced over the years. Bone anchored male sling appears to be effective and safe in intermediate term follow up. It certainly more effective than collagen implant and may provide alternative treatment option in patient with mild to moderate incontinence. In short term, other novel procedures seem to be promising. In spite of new technology, artificial urinary sphincter continues to provide high patient satisfaction and cure rates.

Incontinence following prostatectomy is a devastating complication associated with significant alteration in quality of life. The incidence of urinary incontinence following radical retropubic prostatectomy (RRP), reported in the literature, varies from 2.5-87%.[1] In more recent series, incidences tend to be lower, 2-10%.[2] This can also occur in 1% of patients undergoing surgical treatment for benign prostatic hypertrophy.[1] Although the incidence of postprostatectomy incontinence has decreased with better understanding of the neurovascular bundles and modification of the operative technique, it continues to be one of the most feared complications after surgery. The reason for the wide range in incidence is the use of different definitions of continence and the method of assessment.

Bothersome incontinence causes approximately 10% of patients to seek treatment for their incontinence following radical prostatectomy.[3]

Prostate cancer screening and early detection of prostate cancer has lead to a dramatic increase in the numbers of radical prostatectomy. As more young patients are undergoing radical prostatectomy, the impact of urinary incontinence on the quality of life following surgery assumes greater importance. Herr reported that 26% of men who underwent radical prostatectomy and suffered from urinary incontinence were extremely upset and limited their daily activities.[4]

## PATHOPHYSIOLOGY

Postprostatectomy incontinence (PPI) may be caused by sphincter dysfunction or bladder dysfunction. In recent studies, sphincter weakness was assumed to be the most important reason for incontinence.[2]

Preservation of the functional integrity of the distal urethral sphincter mechanism is germane for maintaining continence postoperatively. Direct surgical injury to the rhabdosphincter or its innervation is often responsible for postoperative incontinence.

Sphincter denervation can also occur as a result of radical pelvic surgery, i.e., abdomino perineal resection, resection of benign prostatic adenoma, pelvic trauma, pelvic irradiation or neurological injury.

During transurethral resection of the prostate (TURP), it is optimal to avoid resecting distal to the verumontanum which represents the most proximal part of the rhabdosphincter. Damage to the sphincter during TURP occurs more commonly anteriorly where the landmark of veru is not visible.

Mostwin suggested several mechanisms of sphincteric injury during or after radical prostatectomy: ischemia and immobilization by scar, atrophy, direct pudendal nerve injury or shortening of the urethra below critical functional length.[5] Several reports have suggested that continent patients have longer functional urethral length than incontinent patients following radical prostatectomy. Rudy *et al* postulated that preservation of continence following radical prostatectomy requires a functional urethral length of at least 2.8 cm.[6] Constantinou and Freiha, however, found no statistical difference in maximum urethral closure pressure or functional urethral length in 13 patients measured pre- and postoperatively.[7]

Various technical modifications have been proposed to preserve as much external sphincter as possible after radical prostatectomy. Walsh *et al*[8] described a modified apical dissection that may lead to earlier continence by incorporating the tissue posterior to the urethra in the viscourethral anastomosis. According to Walsh *et al*, anatomic factors rather than preservation of autonomic innervation are the major factors responsible for improved continence associated with an anatomic approach to radical prostatectomy. They suggested that radiation therapy may induce sphincteric damage or denervation in men, with subsequent sphincteric compromise after radical retropubic prostatectomy.

Detrusor overactivity or loss of bladder compliance is demonstrated during urodynamics in up to 60% of incontinent patients after radical prostatectomy.[9] The associated bladder dysfunction is often a contributing factor, resulting in urinary frequency or urge incontinence and should be treated before surgical management of sphincteric incompetence. Leach *et al* have shown that bladder hyperactivity, demonstrated in patients after radical prostatectomy, often arises *de novo*. They have also speculated that bladder overactivity may be a result of denervation of the bladder base during surgery.[9]

The pathophysiology of incontinence after radical prostatectomy was assessed by Groutz *et al*.[10] The authors examined the various mechanisms of incontinence in 83 men using a combination of clinical and urodynamic parameters, including history, voiding diary, pad test, sophisticated video urodynamics and pressure-flow studies. Intrinsic sphincter deficiency was the most common urodynamic finding and dominant cause of incontinence, occurring in 73 patients (88%). Bladder overactivity, demonstrated in 28 cases (33.7%), was the only urodynamic finding in three patients (3.6%) and was determined to be the main cause of incontinence in six (7.2%). In addition, the authors concluded that low urethral compliance, presumably from urethral scarring, was a significant cause of intrinsic sphincter deficiency in 25 patients (30.1%). Overflow incontinence is most commonly due to a bladder neck contracture should also be ruled out in all patients with post prostatectomy incontinence.

## PATIENT EVALUATION

The evaluation of patients with PPI should begin with a comprehensive history which should include the onset, duration, evolution and the cause of the leakage and the number of pads used. It is important to assess how the incontinence affects the daily activities and if the patient is bothered by the incontinence. The pad weight test may be used to assess the severity of the incontinence objectively. Any history of surgery or radiation should be noted. A voiding diary can be helpful to get the exact quantification of the fluid intake and functional bladder capacity.

### Physical examination

The physical examination is performed with emphasis on the neurological examination assessing the S2-S4 spinal segments, including anal sphincter tone, perineal sensation in S2-S4 segments and bulbo cavernosus reflex. The abdominal examination is performed to detect a distended bladder with overflow incontinence.

### Urodynamic evaluation

The main role of urodynamic evaluation is to differentiate the various causes of PPI and especially to rule out poor bladder compliance, high pressure detrusor over-activity during filling and to rule out any bladder obstruction during the pressure flow study. An assessment of urodynamic bladder capacity is also obtained as most patients with severe incontinence have low functional bladder capacity because of the poor storage.

To evaluate the role of valsalva leak point pressure (VLPP) pressure in predicting the degree of urinary incontinence, Walker *et al* prospectively evaluated 14 patients complaining of PPI. The authors failed to find a correlation between the VLPP pressure and severity of urinary incontinence.[11]

## MANAGEMENT

Incontinence related to documented bladder dysfunction is best treated with fluid restriction and pharmacological therapies. In cases of mixed incontinence, if the urge is the major component it should be treated prior to any treatment directed toward stress urinary incontinence.

### Sphincteric incompetence

The primary management of sphincteric incompetence after radical prostatectomy is bulking agents, artificial urinary sphincter and bulbourethral sling procedures. Spontaneous improvement of urinary incontinence may take up to 12 months. Therefore, it has been recommended that surgical intervention be postponed in men with PPI for at least 12 months.[1] While pelvic floor exercise training and therapy instituted prior to radical prostatectomy aids in the earlier achievement of urinary incontinence, the value of the various approaches to conservative management of PPI generally remains uncertain.[12]

### Bulking agents

Since its introduction in 1993, bovine glutaraldehyde cross-linked (GAX) collagen (Contigen; CR Bard, Covington, GA) has been used extensively as a bulking agent in the treatment of intrinsic sphincter deficiency in men. Enthusiasm for this endoscopic procedure has waned because of the low success rates associated with the procedure and the need for multiple treatments. In short-term studies, significant improvement or cure was achieved in approximately 20-62% of patients,[13] but success rates declined dramatically with longer-term follow-up. In most studies, the average number of treatments ranged from 2.5 to 4.5 and 25 to 35 mL of collagen was commonly used. Bevan-Thomas *et al*[14] reviewed the results of 257 patients with PPI who were treated with collagen and observed for a mean of 28 months. The authors found that 20% of the patients were dry and an additional 39% were significantly improved. The mean number of injections required was 4.4 (range 1 to 11) and the average total amount of collagen used was 36.6 mL.

Traditionally, collagen implant which is well tolerated and has low complication rate, has been recommended for mild to moderate incontinence in male stress urinary incontinence. It has been reported that the best results can be obtained in patients with mild degrees of incontinence and with a preoperative VLPP greater that 60 cmH_2_O.[15] Aboseif *et al*,[16] reported treatment results in 88 patients with collagen injection. Forty-eight per cent of the patients in their series were dry and 22% showed significant improvement. However, clinical results regarding the efficacy of collagen injection are not consistent. Griebling *et al*[17] treated 25 men with incontinence after RRP and TURP and obtained minimal improvement and significant improvement in eight (32%) and two (8%) patients, respectively. Our results were found consistent with the latter studies and only 30% of patients that were cured had significant improvement at a median follow-up of 15 months.[18]

### Male sling

Before the artificial urinary sphincter was introduced, a variety of urethral compression procedures were implied in an attempt to control urinary incontinence. Most notable were the Kaufman procedures which included a crural crossover (Kaufman 1)[19] and then modified to use a synthetic mesh tape that brings crural together in midline (Kaufman 2).[20] A silicone gel device attached to the corpra cavernosa that compresses the ventral urethra. Based on the Kaufman principles, Clemens *et al*[21] reported a bulbourethral sling procedure in 64 men with severe PPI. With a series of tetra flurol ethylene bolsters placed beneath the bulbar urethra through which a suture is passed and then transferred suprapubically using stamey needle lateral to the urethra and bladder neck and the compression of the bulbo urethra is provided. Their goal was an intrapreoperative leak point pressure greater than 150 cm of water. At a mean follow-up of 18 months, 56% of patients became dry and 8% were significantly improved. However, despite the excellent results, sling revision was required in 21% of patients and bolster removal was necessary secondary to infection in 6%. Moreover, 52% of patients had perineal numbness or pain with 26% rating this problem as moderate or severe. This discomfort is most likely due to the high pressure entrapment of pudendal nerve branches during blind suprapubic suture or passage.

More recently, bone-anchored perineal male sling was introduced by Franco and Baum,[22] Madjar *et al*.[23] This was later popularized by Comiter[24] and Rajpurkar.[25] The use of bone anchors obviates the need for blind transfer of sutures suprapubically to achieve bulbourethral compression and eliminates any abdominal incision. It utilizes six 5 mm titanium screws which are drilled into the anteromedial aspects of each descending pubic rami using the InVance bone drill (American Medical Systems). These screws are preloaded with a pair of number 1 polypropylene sutures. The proximal or the topmost bone screws are placed just beneath the junction of the descending ramus and pubic symphysis and the remaining sutures are placed a centimeter apart on each side. A 4 × 7 cm polypropylene mesh alone or in combination with the dermis as a composite graft are used as a sling material. After one side of sling is anchored to the pubic ramus, sling tension is adjusted, either by retrograde leak point pressure method or if the patient is awake, by simple cough method. Sling is then tied down to the opposite pubic ramus.

Unlike the artificial urinary sphincter (AUS) that compresses the urethra circumferentially, thereby interfering with the venous blood flow and predisposing to urethral atrophy and even erosion, the male sling compresses only the ventral aspect of the bulba urethra leaving the dorsal and lateral blood flow intact. Moreover, tissue including the bulbo spongiosus muscle is left intact over the urethra serving as a cushion between the urethra and the sling further minimizing the risk of erosion. The infection and erosion for perineal sling is low (2.1%) and the need for revision caused by bone anchor dislodgement is 4.2%.[26]

Unlike the AUS, the perineal male sling has the advantage of allowing spontaneous physiological voiding without need for manipulation. Excellent cure rates have been reported with the bone-anchored perineal sling and they generally range between 70-90% depending on the method of evaluation and definition of success.[22-25] Comiter recently reported intermediate term results with median follow-up of 48 months (range of 24 to 60 months) The mean pad usage decreased from 4.6 ± 2.1 pads per day to 1.0 ± 1.7 pads per day (*P*<0.01). In total, 65% were considered cured of their leakage and another 15% were significantly improved.[26] Similar results were obtained at the author's institution. Patient satisfaction rate of 70% with a mean follow-up of 24 months and a success rate of 74% were reported.[25]

As more and more experience is gained with this procedure, the author emphasized the importance of patient selection as well as material selection for this procedure which greatly impacted the male sling outcome. In a study of 46 men at a mean follow-up of 18 months, the procedure was found to be successful in 76% and improved in another 35%. Twenty-four per cent of patients failed the procedure and all the failures were found to be due to absorbable graft material. The success rates were significantly greater in patients receiving synthetic mesh, either alone or as composite graft compared with the use of absorbable material alone (75% and 97% versus 0% respectively, *P* value <0.05).[27] It was also found that the patient with mild to moderate incontinence (less than five pads) had a significantly better outcome compared with those with severe incontinence, (five or more pads). The sling failure correlated well with the type of material and severity of the incontinence. Since the introduction of this procedure, it is now established that this procedure is suited for mild to moderate incontinence.

**Figure 1:**
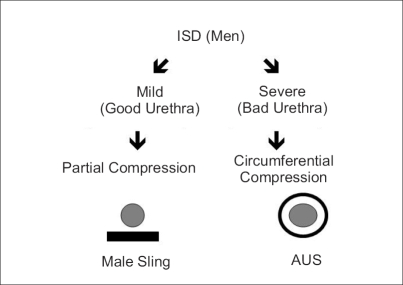
Which procedure to do

The author believes that the partial compression on the ventral aspect of the urethra by male sling is adequate for continence in patients with mild to moderate incontinence as they have an adequate sphincter function but in patients with severe incontinence, with more severe damage to their sphincter mechanism, it requires circumferential compression by artificial urinary sphincter [Figure 1].

On comparing male sling with collagen injection, the authors have found male sling to be more effective than the collagen implant in the treatment of mild to moderate incontinence, 76% versus 30% respectively. Mean number of collagen injections were found to be 2.1 with a range of 1-5 with a mean of 8.8cc (2 to 34cc) collagen injected in 34 patients and another 37 patients received the perineal bone-anchored male sling. There was a statistically significant difference between the two groups, (*P*< 0.05).[18]

In another study, comparing the bone-anchored male sling with artificial urinary sphincter, at a mean follow-up of 22 months, male sling provided comparable efficacy in mild to moderate incontinence as compared to artificial urinary sphincter (90% versus 80% respectively). On the other hand, artificial urinary sphincter was much more superior in patients with severe incontinence, 72% versus 58% respectively.[28] It was concluded that patients with mild to moderate incontinence can be counseled to have equally effective outcomes undergoing male sling as well as artificial urinary sphincter.

**Figure 2:**
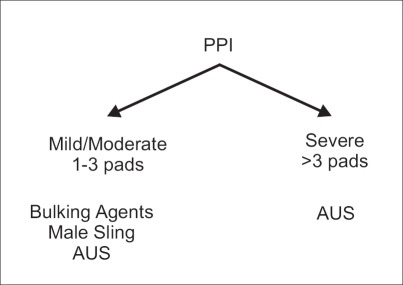
How do we select

Another advantage of male sling would be that it does not preclude AUS implantation at a later date. This observation was obtained from another study looking at feasibility of AUS after the failure of male sling surgery.[29] A total of 18 patients failed the procedure at a mean follow-up of 13 months. Of these, 11 patients proceeded to undergo AUS placement. No complication was encountered during urethral dissection in patients who had prior male sling procedures. A dry rate of 72.7% was found following AUS implantation. And another 9.1% improved in their incontinence. Mean follow-up after salvage AUS was 14.2 months with a range of six to 20 months. Patient satisfaction after AUS placement was 74.5%. It was concluded that AUS placement after a failed bone-anchored male sling is technically feasible and does not affect the short-term efficacy of artificial sphincter. These results were found to be comparable with naïve AUS placement. The algorithm currently followed for the management of PPI by the author is given in Figure 2.

## AUS

AUS is the gold standard treatment for stress urinary incontinence after prostatectomy offering patients the greatest chance of cure. The device provides acceptable social continence in approximately 90% of the patients. First introduced over 30 years ago, the device has continually evolved to its current and sophisticated form. It is widely used in clinical practice and now 100,000 devices have been implanted worldwide. The first commercially available artificial urinary sphincter AMS 721 was introduced by Brantley Scott in 1974.[30] This device has undergone multiple modifications and refinement to its current model AMS 800. This device is an inflatable cuff placed around the urethra or bladder neck, a reservoir placed in the retropubic space and pump placed in the scrotum. All these components are connected by silicone tubing. Components of AUS are filled with normal saline. Pressure within the device can be maintained with a predetermined range, the most common being 61 to 70 cm of water pressure. Cuff lengths also vary from 4 cm to 11 cm to accommodate the urethra and bladder neck dimensions. The device is placed around the bulbar urethra transperineally. More recently, a high transcrotal approach has been advocated.[31]

A large amount of data relating to incontinence rates, complication and patient satisfaction following AUS implantation has accrued since introduction of the device. The largest reported data was 323 patients who received an AUS from the Mayo Clinic.[32] With a mean follow-up of 68.8 months, continence rates of 79% were reported. Recent studies which included all patients with PPI in whom more than half had RP, quoted ‘dry’ rates of 44-86% and a ‘significantly improved’ rate of 90-96%. Patient satisfaction was approximately 90%.[33] The wide range of some of these values can be explained by different definitions of continence and methods of assessment.

In the largest study to date, Elliott and Barrett reported 245 of 271 (90%) patients having a functioning AUS at mean follow-up of five years and 72% required no revision.[32] Another study with 61 patients and a 10-year follow-up reported a 75% continence rate, with 80% of patients having had at least one revision procedure by 10 years.[34] In a comparative study of collagen injection and artificial urinary sphincter by Dimitri *et al*, patients receiving an AUS have a higher quality of life and are more likely to be continent than a patient treated with a collagen injection at a mean follow-up of 19 months. Twenty per cent of collagen patients versus 75% AUS patients were either completely dry or socially continent (*P*< 0.001).[35]

There are several complications that may occur in patients undergoing AUS. The most feared complication is urethral erosion and infection. The current erosion rate is 1-3%.[32,33] This can be precipitated by infection, excessive cuff pressure, decreased vascularity from previous radiation and undersized cuff or trauma from a catheterization through an activated cuff. The infection can be expected to be between 1.8-10%, mean is 3.4%.[36] With the currently available AMS 800, the revision rate runs around 9% and the expected five-year survival of narrow-backed AUS is 75%.[36] Urethral atrophy is a relatively common cause of recurrent incontinence with an incidence of 3-9%. Current management options include downsizing the cuff or placing a tandem cuff.[33] Persistence stress incontinence may occur in up to 15% of patients after AUS insertion.[37] It could be due to too loose a cuff or too low reservoir pressure.

## NEW DEVELOPMENTS

Recently, various adjustable male slings have been introduced in Europe which can be adjusted according to patient needs and recurrence of urinary incontinence, such as ProACT,[37] Reemix[38] and Argus.[39] A new transobturator male sling system (AdVance, American Medical Systems) has been approved by the Food and Drug Administration in the United States. The results were presented by Dr. Rehder at the American Urology Association meeting in Atlanta, GA, 2006.[40] The sling is placed through the transobturator approach which is fixed to the bulbar urethra. The procedure performed in 18 patients was found to be successful in treating the urinary incontinence and all patients reported a decrease in pad usage in the early postoperative follow-up.

## CONCLUSION

Most patients with postprostatectomy incontinence have stress urinary incontinence secondary to intrinsic sphincter deficiency. Patients with bladder dysfunction should be diagnosed and treated prior to surgical intervention. The artificial urinary sphincter is the gold standard in treating patients with stress incontinence. Collagen injection therapy plays a very limited role in the postprostatectomy incontinent patients. Various bulbourethral slings, especially bone-anchored male sling and new devices provide an alternative therapy to the artificial urinary sphincter.

## References

[1] Haab F, Yamaguchi R, Leach GE (1996). Postprostatectomy incontinence. Urol Clin North Am.

[2] Majoros A, Bach D, Keszthelyi A, Hamvas A, Romics I (2006). Urinary incontinence and voiding dysfunction after radical retropubic prostatectomy (prospective urodynamic study). Neurourol Urodynam.

[3] Stanford JL, Feng Z, Hamilton AS, Gilliland FD, Stephenson RA, Eley JW (2000). Urinary and sexual function after radical prostatectomy for clinically localized prostate cancer: The Prostate Cancer Outcomes Study. JAMA.

[4] Herr HW (1994). Quality of life of incontinent men after radical prostatectomy. J Urol.

[5] Mostwin JL (1995). Urinary incontinence. J Urol.

[6] Rudy DC, Woodside JR, Crawford ED (1984). Urodynamic evaluation of incontinence in patients undergoing modified Campbell radical retropubic prostatectomy: A prospective study. J Urol.

[7] Constantinou LE, Freiha FS (1992). Impact of radical prostatectomy on the characteristics of bladder and urethra. J Urol.

[8] Walsh PC, Partin AW, Epstein JI (1994). Cancer control and quality of life following anatomical radical retropubic prostatectomy: Results at 10 years. J Urol.

[9] Leach GE, Trockman B, Wong A, Hamilton J, Haab F, Zimmern PE (1996). Post-prostatectomy incontinence urodynamic findings and treatment outcomes. J Urol.

[10] Groutz A, Blaivas JG, Chaikin DC, Weiss JP, Verhaaren M (2000). The pathophysiology of post-radical prostatectomy incontinence: A clinical and video urodynamics study. J Urol.

[11] Walker C, Mason D, Joseph A, Juma S (1995). Does the leak point pressure predict the severity of urinary incontinence in the male patients?. J Urol.

[12] Parekh AR, Feng MI, Kirages D, Bremner H, Kaswick J, Aboseif S (2003). The role of pelvic floor exercises on post-prostatectomy incontinence. J Urol.

[13] Faerber GJ, Richardson TD (1997). Long-term results of transurethral collagen injection in men with intrinsic sphincter deficiency. J Endourol.

[14] Bevan-Thomas R, Wesley OL, Cespedes RD, McGuire EJ (1999). Long-term follow-up of periurethral collagen injection for male intrinsic deficiency. J Urol.

[15] Sanchez-Ortiz RF, Broderick GA, Chaikin DC, Malkowicz SB, Van Arsdalen K, Blander DS (1997). Collagen injection therapy for post-radical retropubic prostatectomy incontinence: Role of valsalva leak point pressure. J Urol.

[16] Aboseif SR, O'Connell HE, Usui A, McGuire EJ (1996). Collagen injection for intrinsic sphincter deficiency in men. J Urol.

[17] Griebling TL, Kreder KJ, Williams RD (1997). Transurethral collagen injection for treatment of post prostatectomy incontinence in men. Urology.

[18] Onur R, Singla A (2006). Comparison of bone anchored male sling and collagen implant for the treatment of male incontinence. Int J Urol.

[19] Kaufman JJ (1972). Surgical treatment of post-prostatectomy incontinence: Use of the penile crura to compress the bulbous urethra. J Urol.

[20] Kaufman JJ (1973). Treatment of post-prostatectomy urinary incontinence using a silicone gel prosthesis. Br J Urol.

[21] Clemens JQ, Bushman W, Schaeffer AJ (1999). Questionnaire based results of the bulbourethral sling procedure. J Urol.

[22] Franco N, Baum N (2001). Suburethral sling for male urinary incontinence. Infect Urol.

[23] Madjar S, Jacoby K, Giberti C, Wald M, Halachmi S, Issaq E (2001). Bone anchored sling for the treatment of post-prostatectomy incontinence. J Urol.

[24] Comiter CV (2002). The male sling for stress urinary incontinence: A prospective study. J Urol.

[25] Rajpurkar AD, Onur R, Singla A (2005). Patient satisfaction and clinical efficacy of the new perineal bone-anchored male sling. Eur Urol.

[26] Comiter CV (2005). The male perineal sling: Intermediate-term results. Neurourol Urodyn.

[27] Onur R, Rajpurkar A, Singla A (2004). New perineal bone-anchored male sling: Lessons learned. Urology.

[28] Samli M, Singla A, Aggarwal N (2005). Artificial urinary sphincter versus bone anchored male swing for post-radical prostatectomy urinary incontinence. Eur Urol.

[29] Broghammer J, Singla A Feasibility of artificial urinary sphincter after male sling failure. Presented at society for urodynamics and female urology meeting 2006 in Bahamas.

[30] Scott FB, Bradley WE, Timm GW (1974). Treatment of urinary incontinence by an implantable prosthetic urinary sphincter. J Urol.

[31] Wilson SK, Delk JR, Henry GD, Siegel AL (2003). New surgical technique for sphincter urinary control system using upper transverse scrotal incision. J Urol.

[32] Elliott DS, Barrett DM (1998). Mayo Clinic long-term analysis of the functional durability of the AMS 800 artificial urinary sphincter: A review of 323 cases. J Urol.

[33] Litwiller SE, Kim KB, Fone PD, White RW, Stone AR (1996). Post-prostatectomy incontinence and the artificial urinary sphincter: A long-term study of patient satisfaction and criteria for success. J Urol.

[34] Fulford SC, Sutton C, Bales G, Hickling M, Stephenson TP (1997). The fate of the ‘modern’ artificial urinary sphincter with a follow-up of more than 10 years. Br J Urol.

[35] Kuznetsov DD, Kim HL, Patel RV, Steinberg GD, Bales GT (2000). Comparison of artificial urinary sphincter and collagen for the treatment of post prostatectomy incontinence. Urology.

[36] Tse V, Stone AR (2003). Incontinence after prostatectomy: The artificial urinary sphincter. BJU Int.

[37] Hubner WA, Schlarp OM (2005). Treatment of incontinence after prostatectomy using a new minimally invasive device: Adjustable continence therapy. BJU Int.

[38] Sousa-Escandon A, Rodriguez Gomez JI, Uribarri Gonzalez C, Marques-Queimadelos A (2004). Externally re-adjustable sling for treatment of male stress urinary incontinence: Points of technique and preliminary results. J Endourol.

[39] Moreno Sierra J, Victor Romano S, Galante Romo I, Barrera Ortega J, Salinas Casado J, Silmi Moyano A (2006). New male swing “Argus” for the treatment of stress urinary incontinence. Arch Esp Urol.

[40] Rehder P, Gozzi C (2006). A transobturator approach for a sling to treat male urinary incontinence. J Urol.

